# Association between routine laboratory tests and long-term mortality among acutely admitted older medical patients: a cohort study

**DOI:** 10.1186/s12877-017-0434-3

**Published:** 2017-03-01

**Authors:** Henrik Hedegaard Klausen, Janne Petersen, Thomas Bandholm, Helle Gybel Juul-Larsen, Juliette Tavenier, Jesper Eugen-Olsen, Ove Andersen

**Affiliations:** 1 Optimed, Clinical Research Centre, Copenhagen University Hospital, Hvidovre, Denmark; 2 Department of Public Health, Section of Biostatistics, University of Copenhagen, Copenhagen, Denmark; 3 Department of Orthopedic Surgery, Copenhagen University Hospital, Hvidovre, Denmark; 4 Department of Physical Therapy, Physical Medicine & Rehabilitation Research – Copenhagen (PMR-C), Copenhagen University Hospital, Hvidovre, Denmark; 5 The Emergency Department, Copenhagen University Hospital, Hvidovre, Denmark

## Abstract

**Background:**

Older people have the highest incidence of acute medical admissions. Old age and acute hospital admissions are associated with a high risk of adverse health outcomes after discharge, such as reduced physical performance, readmissions and mortality. Hospitalisations in this population are often by acute admission and through the emergency department. This, along with the rapidly increasing proportion of older people, warrants the need for clinically feasible tools that can systematically assess vulnerability in older medical patients upon acute hospital admission. These are essential for prioritising treatment during hospitalisation and after discharge.

Here we explore whether an abbreviated form of the FI-Lab frailty index, calculated as the number of admission laboratory test results outside of the reference interval (FI-OutRef) was associated with long term mortality among acutely admitted older medical patients. Secondly, we investigate other markers of aging (age, total number of chronic diagnoses, new chronic diagnoses, and new acute admissions) and their associations with long-term mortality.

**Methods:**

A cohort study of acutely admitted medical patients aged 65 or older. Survival time within a 3 years post-discharge follow up period was used as the outcome. The associations between the markers and survival time were investigated by Cox regression analyses. For analyses, all markers were grouped by quartiles.

**Results:**

A total of 4,005 patients were included. Among the 3,172 patients without a cancer diagnosis, mortality within 3 years was 39.9%. Univariate and multiple regression analyses for each marker showed that all were significantly associated with post-discharge survival. The changes between the estimates for the FI-OutRef quartiles in the univariate- and the multiple analyses were negligible. Among all the markers investigated, FI-OutRef had the highest hazard ratio of the fourth quartile versus the first quartile: 3.45 (95% CI: 2.83-s4.22, *P* < 0.001).

**Conclusion:**

Among acutely admitted older medical patients, FI-OutRef was strongly associated with long-term mortality. This association was independent of age, sex, and number of chronic diagnoses, new chronic diagnoses, and new acute admissions. Hence FI-OutRef could be a biomarker of advancement of aging within the acute care setting.

**Electronic supplementary material:**

The online version of this article (doi:10.1186/s12877-017-0434-3) contains supplementary material, which is available to authorized users.

## Background

Acutely admitted older medical patients have high risks of low physical reserve capacity, comorbidities, chronic diseases, recurring admissions, and mortality [1–6]. The prevalence of chronic disease increases with age and is associated with poorer functional status, lower quality of life, and increased mortality risk [6]. The rate of new chronic disease also increase with age, and with level of chronic inflammation [7]. Age and elevated inflammation are associated with recurring admissions, frailty, and time to death [8, 9]. In older patients, activities of daily living decline in conjunction with acute hospitalisation, and this decline persists in a majority of patients one year after discharge [10–12]. This may be caused by vulnerability due to advanced biological aging with high accumulation of deficiencies and reduced stability in homeostatic balance, reducing the threshold for coping with stress [9, 13–17].

There is no gold standard for assessing the progression of biological aging or frailty. Long-term mortality – in the sense of time to death not owing to a lethal accident or acute illness, and hence solely reflecting the increasing mortality risk associated with aging – is a feasible way to measure the progression of biological aging and frailty [18, 19]. However, it is not clinically applicable.

A clinically applicable tool to systematically identify patients with advanced biological aging upon admission to an emergency department could help prioritise medical treatment, care, and rehabilitation during hospitalisation and following discharge. To assess the risk of long-term mortality in a clinical setting, biomarkers that reflect the cellular processes of aging at the organism level, across acute illnesses, chronic diseases, and social inequality are needed. For clinical use, biomarker measurement must be simple, reproducible, and able to be performed in an emergency department, from where a substantial number of older patients are discharged [20–22].

Quantification of accumulated deficiencies – by frailty index (FI) [23, 24] and physiological dysregulation -by *D*_*M*_ [17] are comprehensive measures of aging related changes on the organism level. They are based on the concept that assessment of aging needs to reflect the sum of the many different intracellular and intercellular aging changes, which drive aging at the organism level. Variables of aging changes, which are not individually significantly associated with time to death, can be essential for assessing the progression of aging at the organism level. FI is a generic measure as its association with the risk of death, institutionalisation, and worsening health status is reproducible independent of the types of aging-related variables included in the frailty index, especially if 30 variables or more are included [24]. The *D*_*M*_’s association with frailty and mortality is also reproducible with many different clusters of aging related variables [25, 26]. FI is calculated as the number of variables over or under a given deficiency threshold for the specific variable, making FI readily clinical applicable. The *D*_*M*_ being a multivariate statistical distances among sets of biomarkers relatively to the mean of a reference population is more challenging within a clinical setting. The FI have been validated in different populations of mainly community-dwelling individuals [27–31]. One frailty index consisting of common clinical laboratory tests with the addition of blood pressure variables (the laboratory frailty index (FI-Lab)) has to our knowledge not yet been tested within an acute care clinical setting [27, 28, 30]. Acute illness could potentially offset laboratory test results, hence acute illness could be a bias for assessing frailty and advancement of aging by accumulated deficiency in admission laboratory tests. On the other hand, if acknowledging that frailty includes reduced reserve capacity to cope with stress, the number of laboratory test results outside of the reference interval in admission blood samples, could potentially reflect high susceptibility to stress by the acute illness, and hence frailty.

A frailty index composed of 53 variables, of which 18 were the product of common clinical laboratory tests, showed significant association with mortality within 300 days of hospitalisation in older patients in a specialised geriatric intensive care unit [32].

Routine admission laboratory tests from the acute care setting in older medical patients have mainly be tested for their association with —and have acceptable predictive ability for—short-term or in-hospital mortality in conjunction with an acute illness [33–38]. However, their association with long-term mortality is unknown.

We hypothesised that an abbreviated form of FI-Lab - based entirely on standard admission laboratory test results outside of the reference interval (henceforth referred to as FI-OutRef), would be associated with long term mortality in acutely admitted older medical patients.

The objectives of the current study were, first, to explore whether FI-OutRef was associated with long-term mortality risk among acutely admitted older medical patients; and secondly, to investigate how chronological age, the total number of chronic diagnoses, and the numbers of new chronic diagnoses and new acute admissions in the 2-year period preceding admission (henceforth referred to as *age*, *number of chronic diagnoses*, *new chronic diagnoses,* and *new acute admissions*) were associated with survival after discharge. Thirdly, we aimed to compare all five markers of long-term mortality risk.

## Methods

### Design

We conducted a cohort study from administrative and clinical databases. All patients of 65 years of age or older with an acute medical admission to the Emergency Department at Copenhagen University Hospital, Hvidovre, Denmark, between January 1^st^ 2010 and December 31^st^ 2010 were included. The index hospitalisation was defined as the first acute medical admission during this period. Two hospitalisations within 4 hours of each other were defined as one. We collected data regarding in-hospital and out-patient clinical care for 10 years prior to - and 3 years following - the index hospitalisation with a uniform pre- and post-hospitalisation observation period, regardless of the time of admission in the inclusion period. This investigation was approved by the local Danish Data Protection Agency (journal no HVH-2011-14) and the Research Ethics Committees for The Capital Region of Denmark (H-1-2011-FSP). Our reporting of this study adheres to the STrengthening the Reporting of OBservational studies in Epidemiology (STROBE) guidelines, using the checklist for cohort studies [39].

### Setting

Denmark has a public healthcare system that provides all citizens with uniform, feeless, tax-funded treatment for primary medical care, hospital care and homecare services. All Danish citizens have a unique personal identification number, making it possible to track citizens in various national registers.

Acute admission is only provided when the need is determined by a general practitioner, or by ambulance staff following an emergency call. Copenhagen University Hospital, Hvidovre, has a 552-bed capacity, and had 13,024 acute admissions to its emergency department in 2010. For acutely admitted patients, the emergency department performs a standard panel of laboratory tests, including C-reactive protein, leukocytes, differential blood count, haemoglobin, mean corpuscular haemoglobin concentration, mean corpuscular volume, thrombocytes, creatinine, blood urea nitrogen, sodium, potassium, albumin, alanine aminotransferase, alkaline phosphatase, lactate dehydrogenase, bilirubin, and coagulation factors II, VII, and X. When hospitalisation is not required, medical investigation of new symptoms and treatment of chronic diseases are carried out at hospital outpatient clinics by the appropriate medical specialties. The Danish National Patient Register includes information regarding patients’ diagnoses from hospitalisations and outpatient visits, but not from visits to general practitioners. The Danish Civil Registration System provides updated information on vital status by the citizen’s personal identification number. Details on both national registers have previously been provided [40, 41].

### Covariates and outcome variables

We focused on five markers that are strongly or potentially associated with long-term mortality: 1) FI-OutRef, reflecting overall organism dysfunction due to accumulated deficiencies in multiple organ systems; 2) *age*; 3) *number of chronic diagnoses*, reflecting accumulated morbidity; 4) *new chronic diagnoses*, reflecting the rate of comorbidity; and 5) *new acute admissions*, reflecting the severity of disease and impairment.

FI-OutRef was calculated based on the routine panel of 17 laboratory tests undertaken in the emergency department at the index hospitalisation. We identified test results outside of the laboratory’s age- and sex-specific reference intervals (See Additional file 1: Appendix 1 for overview and references). FI-OutRef was calculated for a patient only if data were available for ≥10 of the 17 tests. When data were missing for seven or fewer of the 17 laboratory tests, FI-OutRef was standardised by dividing the number of tests with out-of-reference-interval results by the number of tests performed, and multiplying this number by 17.

The *number of chronic diagnoses* was calculated as the number of unique chronic diagnosis codes during the 10-year period prior to the index hospitalisation. *New chronic diagnoses* were calculated as the number of new unique chronic diagnosis codes recorded for the patient during the 2-year period preceding the index hospitalisation. Chronic diagnoses were identified within all diagnoses from hospitalisations and outpatient clinic visits in the Danish National Patient Register database. A chronic diagnosis was classified using the Healthcare Cost and Utilisation Project’s Chronic Condition Indicator (CCI) tool [42, 43]. The CCI is a dichotomised assessment of United States (US) ICD10 diagnosis codes as chronic or non-chronic, with ‘chronic’ defined as a condition lasting ≥12 months and meeting one or both of the following criteria: “(a) it places limitations on self-care, independent living, and social interactions; (b) it results in the need for ongoing intervention with medical products, services, and special equipment” [42]. To account for differences between the US and Danish ICD10 sub-classifications, an algorithm was developed for identifying chronic diagnoses in the cohort. If the diagnosis code from the CCI classification was identical to the patients’ ICD10 code (only considering number of digits used in the CCI classification), the patients were assigned the same CCI classification. Diagnosis codes that did not match the CCI classification were independently reviewed by two physicians (HHK and OA). We reviewed a list of matching diagnoses between the US ICD10 and Danish ICD10 by the first four characters to find matching specific diagnosis names, and assigned the relevant CCI classification to the Danish ICD10 code. We also reviewed a second list of 450 Danish ICD10 codes, with no matching US ICD10 codes at the four-character level, and classified these codes in accordance with the Healthcare Cost and Utilisation Project’s definition of chronic disease by consensus of the two physicians.

*New acute admissions* were calculated as the accumulated number of acute admissions recorded in the Danish National Patient Register within the 2-year period prior to the index hospitalisation.

Time to death during in-hospital stay and post-discharge were calculated based on the vital status extracted from the Danish Civil Registration System. Charlson Comorbidity Index score was calculated based on all diagnoses registered in the 10-year period prior to the index hospitalisation [44]. Length of stay was defined as the time between admission and discharge. Length of stay was calculated for the index hospitalisation, as well as accumulated for all hospitalisations and for all acute hospitalisations during the 10-year period prior to the index hospitalisation.

### Statistical analysis

Separate analyses were performed for patients with and without a cancer diagnosis within the 10-year period prior to the index hospitalisation, identified by ICD10 codes containing C00–C96. To better compare the results for the five markers, they were each categorised into the nearest possible quartiles.

Cumulative incidence plots were generated to visualise associations between the five markers and post-discharge time to death among non-cancer patients. Censoring was made 3 years after discharge. Cumulative incidence plots were also used to investigate associations between the five markers and in-hospital time to death among non-cancer patients. Discharge was considered a competing event to in-hospital death.

Among non-cancer patients, survival analyses for each of the five markers with regard to post-discharge time to death were carried out using Cox regression analyses. These analyses were performed without adjustment, adjusted for each of the four other markers, and in a fully adjusted model including all five markers and sex. The first quartile of each marker was used as the reference for all analyses.

FI-OutRef had more missing values than the other four markers. Thus, we performed sub-analyses for these four covariates to investigate the effects of potential selection bias. For this, we compared the estimates of the unadjusted analyses of the total cohort to that of the subpopulation with no missing data for FI-OutRef, for each of the other four markers.

To study possible modification effects among the five markers, as well as possible modification effects of sex on the effects of the five markers, we extended the full models for post-discharge time to death to include interactions between pairs of markers, and between sex and each marker.

To make FI-OutRef more clinically applicable, we estimated the optimal cut point for the current data using the Youden index. In a set of secondary analyses, we investigated each laboratory test for potentially carrying the main effect of the association between FI-OutRef and post-discharge time to death. The laboratory tests were ranked according to the disproportion in death 3 years from discharge between patients having the test result inside versus outside the reference interval. FI-OutRef was made a categorical covariate in accordance with the Youden index-identified cut-off level with the highest specificity and sensitivity for predicting time to post-discharge death [45]. Cox regression analyses for time to death were then performed with the categorical FI-OutRef adjusted for each of the seven highest ranking tests of the above-mentioned laboratory tests, as well as with a fully adjusted model in which FI-OutRef was adjusted for all of the seven highest ranking laboratory tests.

For patients with cancer diagnoses, we analysed the associations of the five markers with post-discharge and in-hospital time to death using cumulative incidence plots similar to those constructed for the non-cancer patients.

Statistical analyses were performed using the SAS enterprise guide 6.1 packages (SAS Institute, Cary, NC, USA). Cumulative incidence plots were generated using the analysis package ‘cmprsk’ version 2.2-7 in R Version 3.1.1 (R Foundation, Vienna, Austria).

## Results

### Study flow and patient characteristics

A total of 4,005 patients above 65 years of age were acutely admitted to the Emergency Department at the Copenhagen University Hospital, Hvidovre, in 2010. Of these patients, 833 had a prior or current cancer diagnosis. In-hospital and post-discharge (3-year follow-up) mortality proportions, respectively, were 6.5% and 39.9% among the 3,172 patients without a cancer diagnosis, and 8.8% and 54.9% for the 833 patients with a current or prior cancer diagnosis. Table 1 presents the descriptive data for the non-cancer and cancer patients.

**Table 1:** Characteristics of the study population

	Non-diagnosed cancer					Diagnosed cancer				
Variables	N	Median		IQ		N	Median		IQ	
Sex female (%)	1884	59%				422	51%			
LOS Index (days)	3172	3.0	1.0	-	8.0	833	3.00	1.0	-	8.0
Charlson Score (No.)	3172	1.0	0.0	-	2.0	833	3.00	2.0	-	5.0
CRP (mg/l)	2610	14.0	6.0	-	50.0	714	21.0	6.0	-	68.0
Leukocyte (x10^9^/l)	2548	9.1	7.1	-	12.2	700	9.2	7.1	-	12.4
Neutrophils (x10^9^/l)	2539	6.4	4.6	-	9.4	691	6.6	4.6	-	9.8
Haemoglobin (mmol/l)	2545	7.9	7.1	-	8.6	699	7.5	6.6	-	8.2
MCHC (mmol/l)	2519	20.9	20.4	-	21.5	693	20.8	20.2	-	21.3
MCV (Fl)	2519	90.0	87.0	-	94.0	693	90.0	86.0	-	95.0
Thrombocyte (x10^9^/l)	2540	238.0	190.0	-	302.0	698	246.0	189.0	-	322.0
Creatinine (μmol/l)	2622	86.0	69.0	-	116.0	716	85.0	69.0	-	120.5
BUN (mmol/l)	2562	7.0	5.1	-	10.1	696	7.2	5.1	-	10.9
Sodium (mmol/l)	2622	138.0	135.0	-	141.0	716	138.0	134.0	-	140.0
Potassium (mmol/l)	2594	3.8	3.5	-	4.2	706	3.9	3.5	-	4.3
Albumin (g/l)	2592	36.0	32.0	-	40.0	709	35.0	30.0	-	38.0
ALAT (U/l)	2588	17.0	11.0	-	26.0	708	17.0	11.0	-	26.0
Alkaline Phosphatase (U/l)	2572	80.0	65.0	-	105.0	703	86.0	67.0	-	114.0
LDH (U/l)	2496	182.0	156.0	-	220.0	680	186.0	154.0	-	229.5
Bilirubin (μmol/l)	2591	7.0	5.0	-	11.0	705	7.0	5.0	-	11.0
Factor II, VII, X	2528	0.8	0.6	-	1.0	693	0.8	0.6	-	1.0
**2000-2010**										
Diag. Unique	3172	16.0	9.0	-	25.0	833	22.0	14.0	-	32.0
Outpatient clinic visits	3172	8.0	4.0	-	13.0	833	13.0	8.0	-	19.0
Prior admissions	3172	4.0	1.0	-	7.0	833	6.0	3.0	-	10.0
Prior acute admissions	3172	3.0	1.0	-	6.0	833	4.0	2.0	-	7.0
LOS	3172	19.0	4.0	-	50.0	833	30.0	12.0	-	63.0
LOS acute admissions	3172	12.0	1.0	-	36.0	833	18.0	5.0	-	45.0
**FI-OutRef** (No.)	2607	5.0	3.0	-	8.0	714	6.0	4.0	-	8.5
**Age** (years)	3172	79.4	72.3	-	85.7	833	79.4	73.0	-	85.6
**No. of chronic diag** (No.)	3172	6.0	3.0	-	11.0	833	9.0	5.0	-	13.0
**New chronic diag** (No.)	3172	1.0	0.0	-	3.0	833	2.0	1.0	-	4.0
**New acute admissions** (No.)	3172	1.0	0.0	-	2.0	833	2.0	1.0	-	4.0

*Non-diagnosed cancer* no cancer diagnoses registered within 10 years prior to index admission, *Diagnosed cancer* cancer diagnosis registered within 10 years prior to index admission, *Diag. Unique* the number of unique diagnosis codes, *LOS* length of stay, *CRP* C-reactive protein, *MCHC* mean corpuscular haemoglobin concentration, *MCV* mean corpuscular volume, *BUN* blood urea nitrogen, *ALAT,* alanine aminotransferase, *LDH,* lactate dehydrogenase, *FI-OutRef* number of laboratory tests outside reference interval, *No. of chronic diag* number of chronic diagnoses within the 10 years prior to the index admission, *New chronic diag* number of new chronic diagnoses in the two-year period up to index admission, *New acute admissions* number of new acute admissions in the two-year period up to index admission. Bold fonts indicate variables used as markers of long-term mortality

### Markers and post-discharge mortality among non-cancer patients

**Fig. 1:**
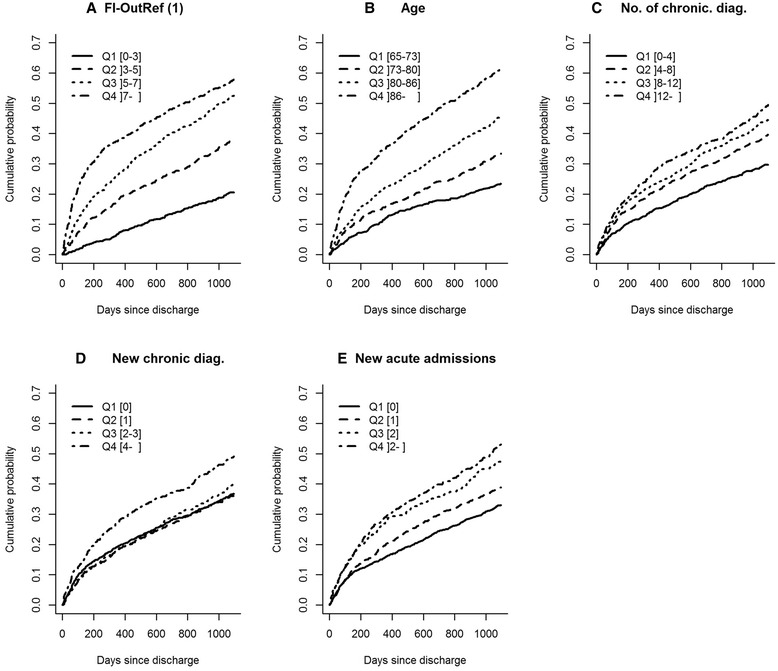
Cumulative incidence plots of mortality within 3 years post-discharge for patients without cancer diagnoses stratified by quartiles (Q1-Q4) of **a**: FI-OutRef, **b**: Age, **c**: No. of chronic diag., **d**: New chronic diag and **e**: New acute admissions. For proportion of cumulative mortality including 95% confidence interval (see Additional file 2: Appendix 2) Abbreviations: FI-OutRef: Frailty index by the number of admission laboratory tests outside the reference interval. No. of chronic diag: number of unique chronic diagnoses within the 10-year period prior to the index admission. New chronic diag: The number of chronic diagnoses given in the 2-year period preceding the index admission. New acute admissions: Number of new acute admissions within the 2-year period preceding the index admission. 1) Note: FI-OutRef includes 2,442 of the 2,965 discharged patients who had 10 or more standard laboratory tests analysed at admission

Visual interpretation of the cumulative incidence plots revealed the clearest separation between quartiles for FI-OutRef and *age* compared with the other markers (Fig. 1a–e). Both were without any overlapping confidence intervals (CI) 3 years post-discharge (See Additional file 2: Appendix 2 for overview of CI of cumulated incidence). For the *number of chronic diagnoses,* the first quartile was the only quartile with no overlapping CI (Fig. 1c). For *new chronic diagnoses*, the fourth quartile had the highest cumulative incidence for death within 3 years post-discharge and was the only quartile with a CI that did not overlap with the nearest quartile’s CI (Fig. 1d). For *new acute admissions,* quartile two and quartile three had non-overlapping CIs, while the CIs overlapped between quartiles one and two, and between quartiles three and four, 3 years post-discharge.

Unadjusted Cox regression analyses showed that all five markers were significantly associated with post-discharge time to death (*P* < 0.001). Table 2 shows the hazard ratios for the investigated markers. Comparing the markers by their hazard ratios for quartiles two to four, relative to quartile one, revealed the strongest association with FI-OutRef, followed by *age.*

**Table 2:** Hazard ratios for time to death post-discharge by quartiles of markers in patients without cancer diagnoses

	Unadjusted^)^		Adjusted										Fully adjusted model	
			FI-OutRef^1^		Age		No. of chronic diagnoses		New chronic diagnoses		New acute admissions			
FI-OutRef^1)^	*P* < .0001				*P* < .0001		*P* < .0001		*P* < .0001		*P* < .0001		*P* < .0001	
Quartile 2	2.09	(1.69–2.58)			1.94	(1.57–2.40)	2.01	(1.62–2.48)	2.05	(1.66–2.54)	2.05	(1.66–2.53)	1.92	(1.55–2.37)
Quartile 3	3.25	(2.65–3.99)			2.84	(2.31–3.49)	3.12	(2.54–3.84)	3.18	(2.60–3.91)	3.18	(2.59–3.90)	2.73	(2.22–3.36)
Quartile 4	4.07	(3.33–4.97)			3.66	(3.00–4.48)	3.85	(3.15–4.71)	4.01	(3.28–4.90)	3.89	(3.18–4.76)	3.48	(2.84–4.26)
Age	*P* < .0001		*P* < .0001				<.0001		*P* < .0001		*P* < .0001		*P* < .0001	
Quartile 2	1.52	(1.26–1.83)	1.41	(1.15–1.74)			1.48	(1.22–1.78)	1.51	(1.25–1.82)	1.51	(1.26–1.82)	1.42	(1.15–1.75)
Quartile 3	2.25	(1.88–2.68)	2.05	(1.69–2.50)			2.13	(1.77–2.53)	2.25	(1.88–2.68)	2.17	(1.82–2.60)	1.99	(1.63–2.42)
Quartile 4	3.60	(3.04–4.27)	3.14	(2.60–3.80)			3.50	(2.95–4.16)	3.69	(3.11–4.38)	3.55	(3.00–4.21)	3.15	(2.60–3.81)
No. of chronic diagnoses	*P* < .0001		*P* < .0001		*P* < .0001				*P* < .0001		*P* < .0001		P 0.07	
Quartile 2	1.42	(1.21–1.68)	1.41	(1.18–1.68)	1.31	(1.11–1.54)			1.45	(1.22–1.72)	1.32	(1.12–1.56)	1.28	(1.06–1.54)
Quartile 3	1.66	(1. 40–1.98)	1.56	(1.28–1.88)	1.50	(1.26–1.79)			1.68	(1.39–2.03)	1.42	(1.18–1.71)	1.32	(1.07–1.63)
Quartile 4	1.93	(1.63–2.27)	1.73	(1.43–2.05)	1.82	(1.54–2.14)			1.87	(1.55–2.26)	1.57	(1.31–1.88)	1.44	(1.17–1.78)
New chronic diagnoses	*P* < .0001		*P* < .0001		*P* < .0001		*P* 0.060				*P* < .0001		P 0.029	
Quartile 2	0.98	(0.83–1.15)	0.95	(0.79–1.13)	0.98	(0.83–1.16)	0.87	(0.74–1.04)			0.84	(0.71–1.00)	0.84	(0.69–1.02)
Quartile 3	1.09	(0.93–1.27)	1.01	(0.85–1.20)	1.07	(0.92–1.25)	0.88	(0.75–1.05)			0.86	(0.72–1.02)	0.81	(0.66–0.98)
Quartile 4	1.48	(1.27–1.71)	1.33	(1.13–1.57)	1.56	(1.35–1.81)	1.07	(0.90–1.27)			1.03	(0.86–1.23)	1.01	(0.82–1.24)
New acute admissions	*P* < .0001		*P* < .0001		*P* < .0001		*P* < .0001		*P* < .0001				*P* <0.001	
Quartile 2	1.23	(1.06–1.43)	1.15	(0.97–1.35)	1.15	(0.99–1.34)	1.12	(0.96–1.31)	1.27	(1.08–1.49)			1.12	(0.94–1.32)
Quartile 3	1.65	(1.39–1.97)	1.35	(1.11–1.64)	1.53	(1.28–1.82)	1.43	(1.19–1.72)	1.69	(1.39–2.04)			1.23	(0.99–1.53)
Quartile 4	1.87	(1.61–2.17)	1.65	(1.40–1.94)	1.82	(1.57–2.11)	1.57	(1.33–1.85)	1.88	(1.58–2.25)			1.49	(1.22–1.81)

Univariate- and multivariate survival analyses regarding mortality within 3 years from discharge in acutely admitted older medical patients by nearest possible quartiles of addressed variables. Columns indicate adjusting covariates in the multivariate survival analysis. Fully adjusted model: adjusted for all of the other addressed covariates and sex. *FI-OutRef* frailty index by the number of laboratory test results outside the reference interval in the standard panel of laboratory tests at admission, *No. of chronic diagnoses* total number of unique chronic diagnoses within the 10-year period prior to the index admission, *New chronic diagnoses*, the number of chronic diagnoses given in the 2-year period up to the index admission, *New acute admissions* number of new acute admissions within the 2-year period up to the index admission 1) Analyses for FI-OutRef includes 2,442 of the total 2,965 discharged patients who had 10 or more standard laboratory tests at admission

When Cox regression analyses were carried out with adjustment in pairs, the hazard ratios for *new chronic diagnoses* were the most altered in comparison with the unadjusted analysis. In the analysis of *new chronic diagnoses* with adjustment for the *number of chronic diagnoses* or *new acute admissions*, the lower limit of the 95% CI of the hazard ratio crossed one for all quartiles. The ranking of the markers according to their hazard ratios between the fourth and the first quartiles did not differ between the fully adjusted models and the unadjusted analyses (Table 2).

Among the 3,172 patients without cancer, 2,607 (≈82%) had data available for 10 or more standard admission laboratory tests. Compared with patients with available FI-OutRef data, those with missing FI-OutRef data had a shorter median length of stay (5.0 versus 5.6 days; Kruskal-Wallis test; *P* < 0.001), a higher proportion of women (54.3% versus 49.7%; chi-square; *P* = 0.007), and a lower median age (78.4 versus 79.4 years; Kruskal-Wallis test; *P* = 0.004). Cumulative incidence of in-hospital and post-discharge death, and mean post-discharge time to death did not significantly differ between the patients with available and those with missing FI-OutRef data. Sub-analyses were performed including only the 2,442 discharged patients with available FI-OutRef data. The results showed negligible changes in the hazard ratios or *P* values for *age*, *number of chronic diagnoses*, *new chronic diagnoses,* or *new acute admissions* compared with the analyses of the full cohort of 2,965 discharged patients (data not shown).

Adjustment for sex did not impact the estimates for any of the markers in the survival analyses regarding post-discharge time to death. Sex was only a significant factor in the survival analysis focusing on *age* (*P* < 0.001). Testing for interactions among the markers with regard to time to death in the fully adjusted model revealed no interactions among the five markers. When testing the modifying effect of sex on each of the markers, the only significant interaction observed was between sex and *new chronic diagnoses* (*P* = 0.02).

**Fig. 2:**
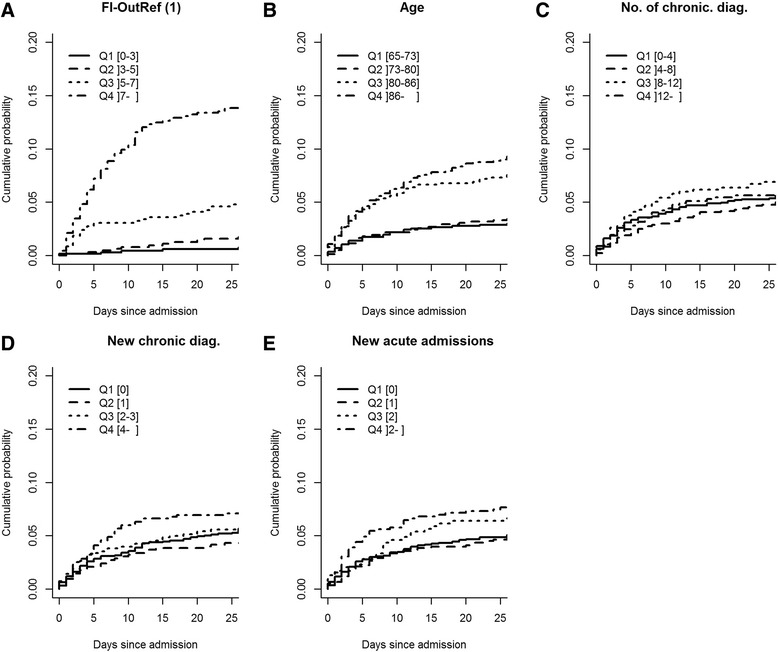
**a** The number (#) of patients with admission laboratory test results outside (out) or inside (in) the reference interval (ref) and their corresponding proportion of mortality within 3 years from discharge. Significance of difference by Chi-Square * ~ *P* ≤ 0.001, ** ~ *P* ≤ 0.05. **b** differences in the mortality proportion for patients inside versus outside the reference interval. FI-OutRef: Frailty index by the number of admission laboratory test results outside the reference interval. MCHC: Mean corpuscular haemoglobin concentration. MCV: Mean corpuscular volume. BUN: Blood urea nitrogen. ALAT: Alanine aminotransferase. LDH: Lactate dehydrogenase. Coag. Fac. II,VII, X: Coagulation factor II, VII and X

Using the Youden index, we determined that an FI-OutRef of ≥5 was the cut-off value with the highest possible sensitivity and specificity for predicting death within 3 years post-discharge. Death proportions 3 years post-discharge were 53.0% for patients with an FI-OutRef of ≥5, and 24.0% for those with an FI-OutRef of <5 (*P* < 0.001) (Fig. 2a). Having an FI-OutRef of ≥5 had a greater impact on post-discharge mortality than having any individual laboratory test result outside of its reference interval (Fig. 2b). The unadjusted survival analysis for post-discharge time to death showed a hazard ratio of 2.9 (95% CI: 2.5–3.3, *P* < 0.001) for having an FI-OutRef of ≥5 compared with <5. When FI-OutRef was adjusted for the seven laboratory tests with the highest discrimination for 3-year mortality (blood urea nitrogen, C-Reactive Protein, alkaline phosphatase, creatinine, neutrophil, albumin, and haemoglobin), FI-OutRef remained significantly associated with time to death (HR 1.5 (95% CI: 1.3–1.9);*P* < 0.001). Blood urea nitrogen had the highest individual impact on the hazard ratio of FI-OutRef, changing the estimate to 2.5 (95% CI: 2.1–2.9, *P* < 0.001).

### Markers and in-hospital mortality among non-cancer patients

**Fig. 3:**
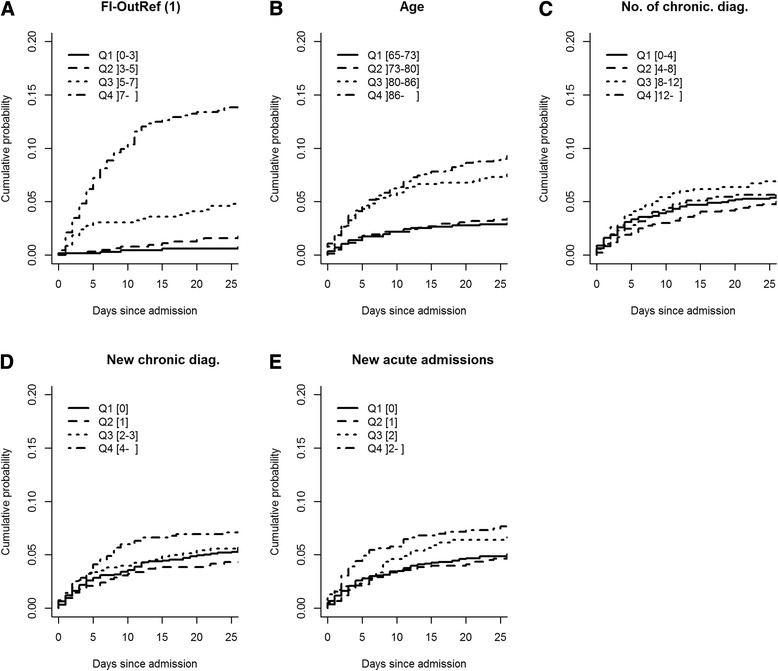
Cumulative incidence plots of mortality for in-hospital mortality for patients without cancer diagnoses stratified by quartiles (Q1-Q4) of **a**: FI-OutRef, **b**: Age, **c**: No. of chronic diag., **d**: New chronic diag and **e**: New acute admissions. For proportion of cumulative mortality including 95% confidence interval (see Additional file 2: Appendix 2). Abbreviations: FI-OutRef: Frailty index by the number of admission laboratory test results outside the reference interval. No. of chronic diag: the number of unique chronic diagnoses within the 10-year period prior to the index admission. New chronic diag: The number of chronic diagnoses given in the 2-year period preceding the index admission. New acute admissions: the number of new acute admissions within the 2-year period preceding the index admission. 1) Note: FI-OutRef includes 2,607 of the 3,172 included patients who had 10 or more standard laboratory tests at admission

For patients without cancer diagnoses, the cumulative incidence plots of death during in-hospital stay (within 3 days post-admission, the time-point at which 50% of patients had been discharged) revealed that FI-OutRef showed the clearest separation among quartiles (Fig. 3a-e). For the cumulative incidence of death for FI-OutRef, the CI for the third quartile overlapped with the CI of the fourth quartile, but not with the CIs of the second or first quartile, which had identical cumulative incidences of death (Fig. 3a) (See Additional file 2: Appendix 2 for overview of CI of cumulated incidence). The cumulative incidences of death within 3 days post-admission for *age*, *number of chronic diagnoses, new chronic diagnoses,* and *new acute admissions* showed no systematic ranking by quartiles, and all CIs were overlapping (Fig. 3b–e).

### Markers and mortality among cancer patients

**Fig. 4:**
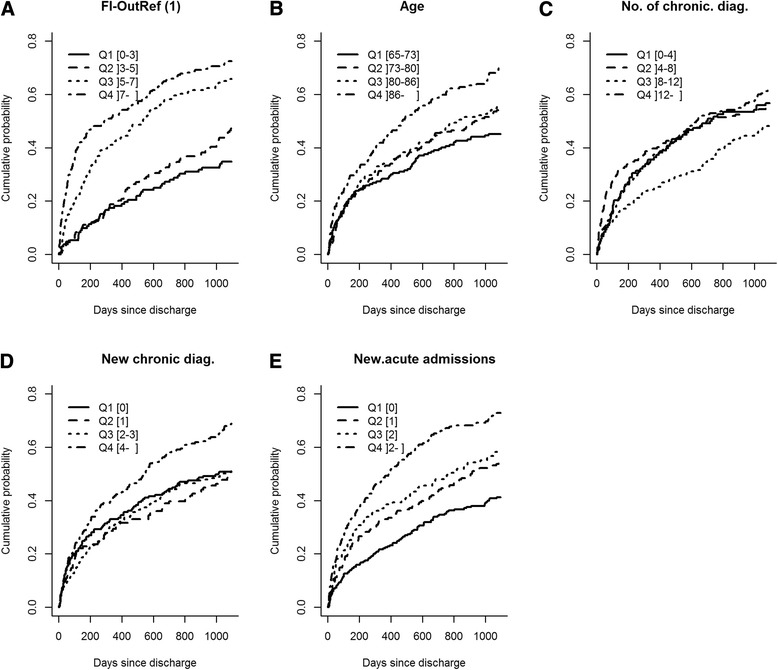
Cumulative incidence plots of mortality within 3 years post-discharge for patients with a cancer diagnoses stratified by quartiles (Q1-Q4) of **a**: FI-OutRef, **b**: Age, **c**: No. of chronic diag., **d**: New chronic diag and **e**: New acute admissions. For proportion of cumulative mortality including 95% confidence interval (see Additional file 2: Appendix 2). Abbreviations: FI-OutRef: Frailty index by the number of admission laboratory test results outside the reference interval. No. of chronic diag: the number of unique chronic diagnoses within the 10-year period prior to the index admission. New chronic diag: the number of chronic diagnoses given in the 2-year period preceding the index admission. New acute admissions: the number of new acute admissions within the 2-year period preceding the index admission. 1) Note: Analyses for FI-OutRef includes 714 of the 726 discharged patients who had 10 or more standard laboratory tests at admission

For post-discharge death among patients with cancer diagnoses only FI-OutRef and *new acute admissions* showed any separation of cumulative incidence curves by quartiles when taking the 95% CI into account (Fig. 4) (See Additional file 2: Appendix 2 for overview of CI of cumulated incidence). Cumulative incidences of death within 3 years post-discharge for the fourth quartiles of FI-OutRef and *new acute admissions* were 71.4% and 72.8%, respectively (Fig. 4a & e). For FI-OutRef, the CIs of cumulative incidence of death within 3 years post-discharge overlapped between the first and the second quartiles, and between the third and the fourth quartiles (Fig. 4a). For *new acute admissions,* only the first quartile had a CI that did not overlap with any of the three other quartiles CI (Fig. 4e).

For the cumulative incidence of death during the in-hospital stay among patients with a cancer diagnosis, none of the five markers showed any discrimination according to quartiles. All CIs for these analyses were overlapping (data not shown).

## Discussion

Our results demonstrate that FI-OutRef was strongly associated with long-term mortality among acutely admitted older medical patients without a cancer diagnosis. This association was independent of sex and of the other investigated markers: *age*, *number of chronic diagnoses*, *new chronic diagnoses,* and *new acute admissions*. Compared with these other markers, FI-OutRef showed the highest hazard ratio for its fourth quartile versus its first quartile, with clear separation from the hazard ratios of the second and third quartiles. *Age* had the second strongest independent association with time to death post-discharge. Among *number of chronic diagnoses* (reflecting accumulated morbidity), *new chronic diagnoses* (reflecting current rate of comorbidity), and *new acute admissions* (reflecting severity of impairment and disease), *new acute admissions* showed the greatest ability to discriminate patients by time to death. With regard to death during in-hospital stay (within 3 days post-admission) among patients with no cancer diagnosis, only the quartiles of FI-OutRef and *age* seemed to discriminate this outcome when evaluated by cumulative incidence plots.

Patients with a cancer diagnosis within the 10-year period prior to the index hospitalisation showed higher rates of overall death within 3 years post-discharge and during their in-hospital stay compared with patients without prior cancer diagnoses. Among patients with a prior cancer diagnosis, none of the investigated potential markers could discriminate patients by cumulative incidence of death 3 days post-admission. In this patient group, only quartiles of FI-OutRef and n*ew acute admissions* could discriminate patients by cumulative incidence of death 3 years post-discharge.

In recent decades, there has been a growing interest in using routine laboratory tests to predict in-hospital mortality among admitted patients, often in combination with age and vital parameters, such as alertness, blood pressure, temperature, heart rate, and respiratory rate [33, 35–37, 46]. Some laboratory tests are already widely used in clinical settings for specific medical conditions, for example, the CURB65 [47] and SAPS II score [48], to assess disease severity in patients with pneumonia and patients in the intensive care unit, respectively.

Within the acute care setting, only few measures have been developed to predict survival after discharge, identifying older medical patients with advanced biological aging. Those present, are often interview-based assessments of activities of daily living, or other measurements of functional inactivity, which carry a risk of patient recall bias [49–52]. Moreover, the above-mentioned tools are time-consuming, and as such, a hindrance for implementation in a time-restrained flow culture such as the emergency department [52, 53]. The frailty index FI-Lab consisting of diastolic- and systolic blood pressure in addition to 21 common laboratory tests, of which 10 were overlapping with the tests in the current study, have so far not been evaluated in older hospitalised patients [27]. In community-dwelling and institutionalised older people, FI-Lab performs as good as of other frailty indices based on clinical and physical performance variables, with regard to long-term mortality risk [27, 28, 30]. These clinical frailty indices were composed of accumulated deficiencies by evaluating variables measuring symptoms, signs, disabilities and diseases.

The markers investigated in the current study are all objective measurements that place no extra strain on emergency department personnel for data collection. The data required to calculate FI-OutRef are usually readily available. When assessing biological aging in an acutely admitted older patient, the patient’s physiological resilience to the stress induced by a given acute disease is an essential indicator of physical reserve capacity—which, in turn, is inversely related to the organ dysfunction induced by biological aging [9, 54]. A laboratory test is typically only used as a clinical predictor of mortality if it is single-handedly significantly associated with —or able to predict— mortality. Thus, a laboratory test is only considered if it reflects a severe single- or multi-organ dysfunction. However, with this practice, we miss the opportunity to detect moderate organ dysfunction that may not impact mortality by itself, but that may have adverse consequences when combined with moderate or severe dysfunction in other organs [17, 24, 28]. Using FI-OutRef to assess overall organism dysfunction improves our estimate of biological aging as well as overall prognosis of the patient. The accumulation of deficiencies in aging, which can be assessed in laboratory tests by FI-Lab in community-dwelling people [27, 28, 30], is highly related to declining recovery time after stress-induced damage [55]. Assessing the advancement of biological aging is an essential supplement to the clinically available organ-specific and disease-specific biomarkers associated with mortality, for example cardiac troponin-tropomyosin in acute myocardial infarction [56, 57]. Such improvement is especially important due to the rapid increase in the population of medical patients above 65 years of age along with their high heterogeneity in multi-morbidity and advancement of biological aging [18, 58].

Few studies have examined how routine laboratory test results are associated with post-discharge mortality in unselected patient cohorts. Walter et al. reported that high creatinine and low albumin at admission were associated with mortality within one year post-discharge in a non-selected group of medical patients over 70 years of age [59]. Among older patients with acute hospitalisations due to heart failure, Novack et al. found that abnormal albumin, blood urea nitrogen, sodium, uric acid, and white blood cell count levels were related to mortality within one year after discharge [60].

In a situation of acute disease or injury, a given laboratory test result can remain within its normal reference interval (homeostatic equilibrium) only if there is sufficient reserve capacity of the involved organs. Compared with individual test results, a high FI-OutRef is likely to reflect a higher number of organs with disturbed homeostatic balance, and thus potentially reflects the overall advancement of the patient’s biological aging. This is supported by the fact that FI-OutRef remained significantly associated with time to death post-discharge in multiple Cox regression analyses, even after adjustment for the seven individual laboratory test results that showed the highest differences in post-discharge proportions of death, being inside versus outside the reference interval.

Among the five investigated markers, FI-OutRef was the only marker with discriminative ability by its quartiles in the cumulative incidence plots of post-discharge death for patients with and without cancer diagnoses. FI-OutRef may be more sensitive for assessing the advancement of biological aging at the organism level compared with *number of chronic diagnoses,* which may reflect organ-specific dysfunction, but with a relatively preserved reserve capacity at the organism level. Moreover, chronic disease documentation may be biased by economic and subjective incentives and, if well treated; chronic disease may have less impact on mortality despite representing organ dysfunction and morbidity. *Age*, regardless of adjustment for the other markers and sex, showed a significant association with post-discharge time to death. The hazard ratios for *age* quartiles were almost of the same magnitude as those for FI-OutRef, indicating aspects of aging beyond those investigated in the current study.

Between the markers of *new chronic diagnoses* and *new acute admissions*, reflecting the rate of morbidity, and disease severity and impairment, respectively, *new acute admissions* had the higher hazard ratio for all of its quartiles with regard to post-discharge time to death among patients without a cancer diagnosis. Aside from FI-OutRef, *new acute admissions* was the only marker that showed any discriminative ability for cumulative incidence of post-discharge death in patients with a cancer diagnosis. As we used death as a surrogate measurement for biological aging, the impact of a *new chronic diagnosis* may have been reduced by relevant medical interventions. Accordingly, the need for acute admission may have better reflected low physical reserve capacity in this non-selected population of patients with acute diseases. This was underlined by the high reduction in the hazard ratio for *new chronic diagnoses* with regard to post-discharge time to death following adjustment for *new acute admissions.*

### Strengths and limitations

This study had several major strengths. First, this was an unselected cohort study with complete follow-up. Second, for each patient, data were collected from 10 years before inclusion until 3 years after discharge. Finally, the study results are highly clinically relevant, as the investigated laboratory tests are objectively assessed as part of routine practice in most emergency departments in developed countries. Thus, implementation of FI-OutRef would not place additional demands on the emergency department’s manpower, and as such, would support equality in healthcare.

Our study also had several limitations. First, the recorded diagnoses were only obtained from hospital admissions and outpatient clinics. Although these would be the diagnoses available to the clinical staff at hospital admission, it is possible that the numbers of diagnoses were underestimated. Second, some patients were missing laboratory test data. However, the results were not altered in the analyses of the four other markers among patients who were not missing FI-OutRef values. Imputation of missing value and latent variable modelling may have improved the laboratory tests’ association with long-term mortality but it would compromise their feasibility in a clinical setting. Third, accidental deaths were not identified, which could bias time to death as a surrogate measurement of biological aging. Even though morbidity and hospitalisation are risk factors of suicide [61], the absolute numbers of suicides and accidents are small, representing 0.09% and 1.7% of the death causes respectively within the age group in Denmark in 2010 [62]. Finally, the only feasible surrogate measurement for biological aging was time to death within 3 years after discharge. It is possible that other measures, such as measures of physical and cognitive function, could better reflect the progression in biological aging and be of greater clinical relevance for rehabilitation and care intensity.

## Conclusions and perspectives

In a population of acutely admitted patients who were 65 years of age or older, we demonstrated that the abbreviated form of FI-lab; FI-OutRef – based on the number of admission laboratory test results outside of the reference interval, was strongly associated with long-term mortality post-discharge. This association was independent of sex and other known or possible prognostic markers of biological aging, including *age*, *number of chronic diagnoses*, *new chronic diagnoses* and *new acute admissions*. Each of the five investigated markers was a significant risk factor associated with time to death within 3 years following discharge, when taking account of sex and the other markers. Among the five markers FI-OutRef showed the highest potential for identifying advanced biological aging, measured as the hazard ratio for post-discharge time to death relative to its quartiles.

The current findings warrant further examination of these long-term mortality markers that can be assessed at low cost and with little manpower. Future studies should investigate their associations with additional measurements related to quality of life and aging, such as mobility, and physical and cognitive performance. Implementation of an objective systematic assessment of biological aging progression within a clinical setting, such as an emergency department, requires determining which clinical assessment tools provide added predictive value, by identifying the smallest cluster of aging-related variables with minimal manpower demands. In line with the existing work of accumulated deficiency and homeostatic physiological dysregulation in aging, these assessment tools need to cover different body systems to potentially assess risk of late-life complications across acute illnesses, chronic diseases, and social inequality, all of which are highly important for optimising treatment and care among the increasing population of older individuals.

## Additional files

Additional file 1: Appendix 1. Reference interval for standard admission laboratory tests. (DOCX 18 kb)
Additional file 2: Appendix 2. Figs. 1, 3 and 4. Cumulated mortality (95% confidence interval) by quartiles of markers. (DOCX 17 kb)

## Abbreviations

CCI: Chronic condition indicator
CI: Confidence interval
FI-OutRef: Frailty index by the number of admission laboratory test results outside of the reference interval
ICD-10: The international classification of diseases, tenth revision
