# Erratum to: Association between routine laboratory tests and long-term mortality among acutely admitted older medical patients: a cohort study

**DOI:** 10.1186/s12877-017-0463-y

**Published:** 2017-03-15

**Authors:** Henrik Hedegaard Klausen, Janne Petersen, Thomas Bandholm, Helle Gybel Juul-Larsen, Juliette Tavenier, Jesper Eugen-Olsen, Ove Andersen

**Affiliations:** 1 Optimed, Clinical Research Centre, Copenhagen University Hospital, Hvidovre, Denmark; 2 Department of Public Health, Section of Biostatistics, University of Copenhagen, Copenhagen, Denmark; 3 Department of Orthopedic Surgery, Copenhagen University Hospital, Hvidovre, Denmark; 4 Department of Physical Therapy, Physical Medicine & Rehabilitation Research – Copenhagen (PMR-C), Copenhagen University Hospital, Hvidovre, Denmark; 5 The Emergency Department, Copenhagen University Hospital, Hvidovre, Denmark

## Erratum

After the publication of this work [1] it was noticed that Fig. 2 is Fig. 3 duplicated. The correct figure is shown below.

**Fig. 2:**
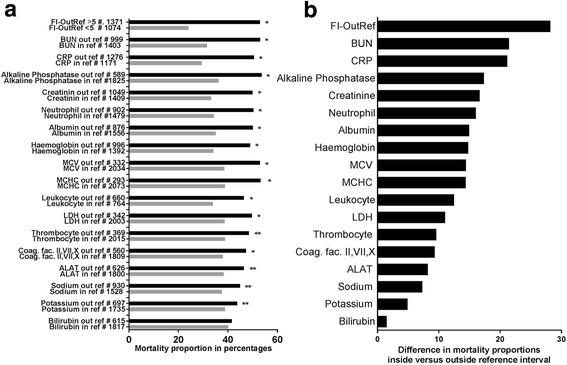
**a** The number (#) of patients with admission laboratory test results outside (out) or inside (in) the reference interval (ref) and their corresponding proportion of mortality within 3 years from discharge. Significance of difference by Chi-Square * ~ *P* ≤ 0.001, ** ~ *P* ≤ 0.05. **b** differences in the mortality proportion for patients inside versus outside the reference interval. FI-OutRef: Frailty index by the number of admission laboratory test results outside the reference interval. MCHC: Mean corpuscular haemoglobin concentration. MCV: Mean corpuscular volume. BUN: Blood urea nitrogen. ALAT: Alanine aminotransferase. LDH: Lactate dehydrogenase. Coag. Fac. II,VII, X: Coagulation factor II, VII and X]
