# Supplementary material for: Association between routine laboratory tests and long-term mortality among acutely admitted older medical patients: a cohort study
Source: BMC Geriatr. 2017 Mar 1;17:62. doi: 10.1186/s12877-017-0434-3 (PMC5333426; doi:10.1186/s12877-017-0434-3)
Supplement: Additional file 1: Appendix 1. — Reference interval for standard admission laboratory tests. (DOCX 18 kb) [file 12877_2017_434_MOESM1_ESM.docx]

Additional file 1: Appendix 2. Reference interval for standard admission laboratory tests.

|  | | | |  |  |
| --- | --- | --- | --- | --- | --- |
| CRP (mg/l)^(1)^ | 0 | - | 10 | |  |
| Leukocyte (x10^9^/l) ^(2)^ | 3.5 | - | 8.8 | | |
| Neutrophils (x10^9^/l)^(3)^ | 1.8 | - | 7.4 | | |
| Hemoglobin (mmol/l) ^(2)^ women | 7.3 | - | 9.5 | | |
| men | 8.3 | - | 10.5 | | |
| MCHC (mmol/l) ^(2)^ | 19.7 | - | 22.2 | | |
| MCV (Fl) ^(2)^ | 82 | - | 98 | | |
| Thrombocyte (x10^9^/l) ^(2)^ | 145 | - | 390 | | |
| Creatinine (µmol/l)^(4)^ women | 50 | - | 90 | | |
| men | 60 | - | 105 | | |
| BUN (mmol/l) ^(4)^ women | 3.1 | - | 7.9 | | |
| men | 3.5 | - | 8.1 | | |
| Sodium (mmol/l) ^(4)^ | 137 | - | 144 | | |
| Potassium (mmol/l) ^(4)^ | 3.5 | - | 4.6 | | |
| Albumin (g/l) ^(4)^ 40–69 years of age | 36 | - | 45 | | |
| 70–125 years of age | 34 | - | 45 | | |
| ALAT (U/l) ^(4)^ women | 10 | - | 45 | | |
| men | 10 | - | 70 | | |
| Alkaline Phosphatase (U/l) ^(4)^ | 35 | - | 105 | | |
| LDH (U/l) ^(4)^ | 115 | - | 255 | | |
| Bilirubin (µmol/l) ^(4)^ | 5 | - | 25 | | |
| Factor II, VII, X ^(5)^ | 0 | - | 0.6 | | |
| **Abbrevations: MCHC:** Mean corpuscular hemoglobin concentration. **MCV:** Mean corpuscular volume.  **BUN:** Blood urea nitrogen. **ALAT:** Alanine aminotransferase. **LDH:** Lactate dehydrogenase.  1. Macy EM, Hayes TE, Tracy RP. Variability in the measurement of C-reactive protein in healthy subjects: implications for reference intervals and epidemiological applications. Clinical Chemistry. 1997;43:52–8.  2. Nordin G, Mårtensson A, Swolin B, Sandberg S, Christensen NJ, Thorsteinsson V, et al. A multicentre study of reference intervals for haemoglobin, basic blood cell counts and erythrocyte indices in the adult population of the Nordic countries. Scandinavian Journal of Clinical and Laboratory Investigation. 2004;64:385–98.  3. d’Onofrio G, Zini G. Morphology of the Blood. Verduci Publisher; 1997.  4. Rustad P, Felding P, Franzson L, Kairisto V, Lahti A, Mårtensson A, et al. The Nordic Reference Interval Project 2000: recommended reference intervals for 25 common biochemical properties. Scandinavian Journal of Clinical and Laboratory Investigation. 2004;64:271–84.  5. Unpublished data from the Copenhagen General Population Study. | | | | | |
