# Supplementary material for: Association between routine laboratory tests and long-term mortality among acutely admitted older medical patients: a cohort study
Source: BMC Geriatr. 2017 Mar 1;17:62. doi: 10.1186/s12877-017-0434-3 (PMC5333426; doi:10.1186/s12877-017-0434-3)
Supplement: Additional file 2: Appendix 2. — Figs. 1, 3 and 4. Cumulated mortality (95% confidence interval) by quartiles of markers. (DOCX 17 kb) [file 12877_2017_434_MOESM2_ESM.docx]

Additional file 2: Appendix 2. Figure S1, S and S4. Cumulated mortality (95 % confidence interval) by quartiles of markers.

| three years from discharge for patient without present or prior cancer diagnosis.  **see figure 1.** | | | | | | | | | | |
| --- | --- | --- | --- | --- | --- | --- | --- | --- | --- | --- |
|  | **FI-OutRef** | | **Age** | | **No. of chronic diag** | | **New chronic diag** | | **New acute admissions** | |
| **Q1** | 20.6% | (17.6 – 23.6%) | 23.4% | (20.5 – 26.3%) | 29.7% | (26.6 – 32.7%) | 36.7% | (33.9 – 39.5%) | 33.0% | (30.5 – 35.5%) |
| **Q2** | 38.1% | (34.2 – 41.9%) | 33.5% | (30.1 – 36.8%) | 39.6% | (36.3 – 42.8%) | 36.3% | (32.3 – 40.2%) | 38.8% | (35.2 – 42.4%) |
| **Q3** | 52.5% | (48.3 – 56.8%) | 45.6% | (42.0 – 49.3%) | 44.4% | (40.3 – 48.5%) | 40.0% | (36.2 – 43.8%) | 48.2% | (43.0 – 53.4%) |
| **Q4** | 57.9% | (53.9 – 61.9%) | 61.4% | (57.8 – 65.1%) | 49.5% | (45.7 – 53.3%) | 49.1% | (45.1 – 53.1%) | 53.1% | (48.8 – 57.3%) |
|  |  |  |  |  |  |  |  |  |  |  |
| three days post-admission, equivalent to 50% of patients had been discharged for patients without present or prior cancer diagnosis.  **see figure 3.** | | | | | | | | | | |
|  |  | |  | |  | |  | |  | |
|  | **FI-OutRef** | | **Age** | | **No. of chronic diag** | | **New chronic diag** | | **New acute admissions** | |
| **Q1** | 0.2% | (-0.2–0.4%) | 1.4% | (0.6–2.2%) | 2.6% | (1.7– 3.5%) | 2.2% | (1.4–3.0%) | 2.1% | (1.3 – 2.8%) |
| **Q2** | 0.2% | (-0.2–0.5%) | 1.1% | (0.4–1.9%) | 1.7% | (0.8 – 2.5%) | 1.8% | (0.7–2.8%) | 1.7% | (0.8 – 2.6%) |
| **Q3** | 2.4% | (1.2–3.6%) | 3.3% | (2.0–4.5%) | 2.8% | (1.4 – 4.2%) | 2.6% | (1.4 – 3.8%) | 2.1% | (0.6 – 3.5%) |
| **Q4** | 4.8% | (3.2–6.4%) | 3.7% | (2.4–5.1%) | 2.3% | (1.1 – 3.5%) | 2.8% | (1.5 – 4.1%) | 3.9% | (2.3 – 5.5%) |
|  |  |  |  |  |  |  |  |  |  |  |
| three years from discharge for patient with a present or prior cancer diagnosis.  **see figure 4.** | | | | | | | | | | |
|  | **FI-OutRef** | | **Age** | | **No. of chronic diag** | | **New chronic diag** | | **New acute admissions** | |
| **Q1** | 34.8% | (26.7 – 43.0%) | 45.2% | (38.2 – 52.2%) | 56.8% | (47.8 – 65.8%) | 50.8% | (43.6 – 58.0%) | 41.2% | (35.0 – 47.4%) |
| **Q2** | 47.2% | (39.0 – 55.4%) | 54.5% | (47.6 – 61.5%) | 55.4% | (48.5 – 62.2%) | 50.0% | (41.6 – 58.4%) | 55.3% | (48.2 – 62.5%) |
| **Q3** | 65.8% | (58.0 – 73.5%) | 55.9% | (48.7 – 63.0%) | 48.2% | (41.1 – 55.3%) | 51.1% | (44.5 – 57.8%) | 59.1% | (50.4 – 67.7%) |
| **Q4** | 72.4% | (66.4 – 78.4%) | 69.8% | (62.9 – 76.7%) | 62.1% | (55.9 – 68.2%) | 68.8% | (62.6 – 75.1%) | 72.8% | (66.5 – 79.1%) |
